# A Ferroptosis-Related Gene Signature for Predicting the Prognosis and Drug Sensitivity of Head and Neck Squamous Cell Carcinoma

**DOI:** 10.3389/fgene.2021.755486

**Published:** 2021-10-21

**Authors:** Wei Lu, Yihua Wu, Shengyun Huang, Dongsheng Zhang

**Affiliations:** ^1^ Department of Oral and Maxillofacial Surgery, Shandong Provincial Hospital, Cheeloo College of Medicine, Shandong University, Jinan, China; ^2^ Department of Oral Medicine, Shandong Provincial Hospital Affiliated to Shandong First Medical University, Jinan, China

**Keywords:** head and neck squamous cell carcinoma (HNSCC), ferroptosis, gene signature, prognosis, drug sensitivity

## Abstract

Head and neck squamous cell carcinoma (HNSCC) is one of the most common cancers worldwide and has a high mortality. Ferroptosis, an iron-dependent form of programmed cell death, plays a crucial role in tumor suppression and chemotherapy resistance in cancer. However, the prognostic and clinical values of ferroptosis-related genes (FRGs) in HNSCC remain to be further explored. In the current study, we constructed a ferroptosis-related prognostic model based on the *Cancer* Genome Atlas database and then explored its prognostic and clinical values in HNSCC via a series of bioinformatics analyses. As a result, we built a four-gene prognostic signature, including *FTH1*, *BNIP3*, *TRIB3*, and *SLC2A3*. Survival analysis showed that the high-risk group presented significantly poorer overall survival than the low-risk group. Moreover, the ferroptosis-related signature was found to be an independent prognostic predictor with high accuracy in survival prediction for HNSCC. According to immunity analyses, we found that the low-risk group had higher anti-tumor immune infiltration cells and higher expression of immune checkpoint molecules and meanwhile corelated more closely with some anti-tumor immune functions. Meanwhile, all the above results were validated in the independent HSNCC cohort GSE65858. Besides, the signature was found to be remarkably correlated with sensitivity of common chemotherapy drugs for HNSCC patients and the expression levels of signature genes were also significantly associated with drug sensitivity to cancer cells. Overall, we built an effective ferroptosis-related prognostic signature, which could predict the prognosis and help clinicians to perform individualized treatment strategy for HNSCC patients.

## Introduction

Head and neck squamous cell carcinoma (HNSCC) is the eighth most common cancer worldwide, accounts for the majority of head and neck cancers and has a high mortality rate of 40–50% (Bray et al., 2018; Moskovitz et al., 2018). Although advances in surgical methods and novel drugs have improved the quality of life of patients with HNSCC, the survival rates have not improved significantly in recent decades (Mannelli et al., 2015). With the aim to solve this issue, many clinical features have been used as prognostic factors to develop efficient and personalized therapeutic strategies. However, some patients with similar clinical characteristics have different prognosis as a result of molecular heterogeneity (Wu et al., 2019). Therefore, it is particularly important to identify a reliable prognosis assessment model which can be used to predict the prognosis of HNSCC patients and to help clinicians develop reasonable therapeutic strategies.

Ferroptosis is a newly discovered form of cell death that is driven by iron-dependent lipid peroxidation and is controlled by numerous metabolic pathways (Liu et al., 2020). Accumulating evidence indicates that ferroptosis is related to tumor suppression and has anti-tumor properties, especially in cases with acquired resistance (Stockwell et al., 2020). Moreover, ferroptosis has been shown to play an important role in the development and treatment of HNSCC. Roh et al. suggested that the induction of ferroptosis via pharmacological and genetic inhibition of cystine/glutamate antiporter can overcome cisplatin resistance of head and neck cancer (Roh et al., 2016). *GLRX5* inhibition can activate the iron responsive element-binding activity of iron regulatory protein, which may upregulate the iron-starvation response, boost intracellular free iron and thus promote ferroptosis (Ye et al., 2010; Lee et al., 2020). Fan et al. indicated that increased *NRF2* could prevent ferroptosis (Fan et al., 2017), and meanwhile some studies showed decreased *NRF2* could enhance the sensitivity of cancer cells to pro-ferroptotic agents (Sun et al., 2016; Roh et al., 2017). Besides, inhibition of *CISD2* can promote sorafenib-induced ferroptosis in resistant cancer cells, and this process promoted excessive iron ion accumulation through autophagy, leading to ferroptosis (Li et al., 2021). Additionally, the suppression of these ferroptosis-related genes (FRGs, such as *GLRX5*, *NRF2*, and *CISD2*) can overcome the resistance to chemotherapy in HNSCC via promoting ferroptosis and may be useful to provide new treatment strategies for patients with drug resistance (Roh et al., 2017; Kim et al., 2018; Lee et al., 2020). Besides, previous studies have shown that some drugs can cause head and neck cancer cell death through inducing ferroptosis (Lin et al., 2016; Kim et al., 2018). However, the prognostic and clinical values of FRGs in HNSCC patients remain unclear.

In this study, we constructed a prognostic signature with four FRGs based on mRNA expression profiles from the Cancer Genome Atlas (TCGA) dataset. Survival analysis and prognostic accuracy analysis of the signature were explored in TCGA-HNSCC cohort and then validated in the independent HNSCC cohort GSE65858. Moreover, the possible signaling pathways, immune correlation and drug sensitivity related to the signature were also analyzed. Overall, our results may provide a novel predictive tool and treatment option for patients with HNSCC.

## Materials and Methods

### Data Collection and Clinical Specimens

The datasets used in the present study are all available on public databases. The RNA-sequencing (RNA-seq) expression data and the corresponding clinical information of HNSCC samples were obtained from the TCGA GDC portal (https://portal.gdc.cancer.gov/repository) and the Gene Expression Omnibus (GEO) database (https://www.ncbi.nlm. nih. gov/geo/). A total of 259 FRGs were downloaded from the ferroptosis database (FerrDb; http://www.zhounan.org/ferrdb) (Zhou and Bao, 2020). The validation data of mRNA expression and DNA copy number was retrieved from the Oncomine database (https://www.Oncomine.org/) (Rhodes et al., 2004). Besides, we also obtained immunohistochemistry (IHC) validation data from the Human Protein Atlas (HPA) database (https://www.proteinatlas.org/).

### Construction of a Ferroptosis-Related Gene Prognostic Signature

Based on the TCGA-HNSCC dataset, the “limma” R package was used to identify the differentially expressed FRGs in HNSCC tissues vs. adjacent non-cancerous tissues via Wilcoxon test, with a false discovery rate (FDR) < 0.01. The Bioconductor packages “clusterProfiler” and “enrichplot” were then used for gene ontology (GO) and Kyoto Encyclopedia of Genes and Genomes (KEGG) enrichment analysis of the differentially expressed FRGs. Meanwhile, univariate Cox analysis of overall survival (OS) was performed to screen prognostic FRGs; the cutoff *p*-value was defined as 0.001. Next, LASSO Cox regression analysis was applied to construct a prognostic signature based on the above genes using the R package “glmnet.” Based on the established risk model, we calculated the risk score of each patient and identified the median risk score of all HNSCC samples. The risk score was calculated as follows: Risk score = $\overset{n}{\underset{j=1}{\sum }}Coefj^{*}Xj$ , where Coefj denotes the regression coefficient and Xj denotes the normalized expression level of each FRG (Miao et al., 2020). The patients were assigned to low or high-risk group according to the median risk score.

### Prognostic Values of the Constructed Prognostic Model

We performed principal-component analysis (PCA) based on the expression profiles of all genes and the prognostic signature genes in both TCGA cohort and GEO cohort using the “stats” R package. Next, we used the “survival” package in R to compare the OS between the low and high-risk groups and to plot Kaplan–Meier survival curves. Univariate and multivariate Cox regression analyses were used to determine whether the risk signature could act as an independent prognostic indicator. Additionally, the time-dependent Receiver Operating Characteristic (ROC) curves of clinical characteristics and the risk signature were drawn with the R package “survival ROC” and the Area Under the Curve (AUC) values at 1 year were calculated. Meanwhile, ROC curves of the risk signature at 1 year, 3 and 5 years were also drawn. The risk signature was then validated in the GSE65858 cohort.

### Construction and Validation of a Predictive Nomogram

In order to predict the prognosis of patients with HNSCC more accurately, we draw a nomogram with age, gender, stage, T stage, N stage, and risk score using “rms” R package. Meanwhile, time-dependent calibration curves were used to evaluate the accuracy of the predictive nomogram at 1, 2 and 3 years.

### Functional Enrichment Analysis of Different Risk Groups

Next, to explore the signaling pathways related to the risk signature, we performed gene set enrichment analysis (GSEA) based on the model gene expression between the low and high-risk subgroups. We set the number of permutations as 1,000 and chose the top five results in each group to build an enrichment plot.

### Tumor Immunity Analysis

The stromal, immune, and ESTIMATE scores were compared between low and high-risk groups with the “ESTIMATE” R package. We then used the single-sample GSEA (ssGSEA) with “GSVA” and “GSEABase” R packages to evaluate some immune-related characteristics (including the infiltrating score of 16 immune cells and the activity of 13 immune-related pathways) between different risk groups (Liang et al., 2020). Meanwhile, the abundance of 22 immune cells was estimated via CIBERSORT algorithm (https://cibersort.stanford.edu/) to further compare the different immune infiltration levels between low and high-risk groups. Besides, we analyzed the different expression levels of immune checkpoints including *PD-1*, *CTLA4*, *LAG3*, *TIGIT*, and *BTLA* between low and high-risk groups.

### Drug Susceptibility Analysis

In order to explore the clinical significance of the constructed prognostic model for HNSCC treatment, “pRRophetic” R package was used to calculate the half-maximal inhibitory concentration (IC50) of common chemotherapeutic drugs in TCGA cohort. According to National Comprehensive *Cancer* Network (NCCN) guidelines Version 2.2021, Cisplatin, Paclitaxel, Docetaxel, Doxorubicin, Etoposide, Gemcitabine, Methotrexate, and Cytarabine were main chemotherapeutic agents for head and neck cancers. Besides, on the basis of previous studies (Tang et al., 2019; Gulati et al., 2020), IC50 of Gefitinib and Metformin was also explored in different risk groups. Then, we analyzed the correlations between the expression of prognostic risk genes and the resistance/sensitivity of pan-cancer cells to chemotherapeutic drugs based on the CellMiner database (https://discover.nci.nih.gov/cellminer), which is an open-access Web interface containing molecular and pharmacological data for the NCI-60 cancerous cell lines (a panel of 60 diverse human cancer cell lines) (Reinhold et al., 2012). And we totally chose 218 drugs approved by FDA from this database.

### Statistical Analysis

All statistical analyses were performed using R software (version 4.0.3; https://www.R-project.org) and Perl software (version 5.32.0.1-64bit; https://strawberryperl.com/). In this study, *p* < 0.05 was known as “statistically significant”, *p* < 0.01 was regarded as “more statistically significant” and *p* < 0.001 was taken as “most statistically significant”.

## Results

### Identification of HNSCC Samples and FRGs

A total of 545 HNSCC samples, including 501 tumor samples and 44 normal samples, from the TCGA dataset were enrolled. Meanwhile, RNA-seq and clinical data of 270 HNSCC samples from GEO-HNSCC cohort was used as the validation dataset. Patients with a follow-up time <60 days and those with no survival information were excluded. Finally, 486 patients from TCGA-HNSCC cohort and 266 patients from GEO-HNSCC cohort were included in the analyses, the detailed clinical characteristics of whom were listed in Table 1. Besides, among 259 FRGs, 45 FRGs that were just tested in non-human species were excluded and a total of 187 genes were identified in the above two cohorts.

**TABLE 1:** Clinical characteristics of HNSCC patients from TCGA and GEO datasets in the study.

Variable	No. of samples in TCGA	No. of samples in GEO
Gender		
Male/Female	359/127	219/47
Age at diagnosis		
≤60/>60	241/245	151/115
Tumor grade		
G1-2/G3-4/unknown	350/117/19	NA
Clinical stage		
I-II/III-IV/unknown	93/326/67	54/212/0
T stage		
T0-2/T3-4	173/259/54	114/112/0
M stage		
M0/M1/unknown	178/1/307	NA
N stage		
N0/N1-3/unknown	164/230/92	92/174/0

HNSCC, head and neck squamous cell carcinoma; TCGA, the *Cancer* Genome Atlas.

GEO, Gene Expression Omnibus; NA, Not Available.

### Identification of Differentially Expressed FRGs With Prognostic Value

**FIGURE 1:**
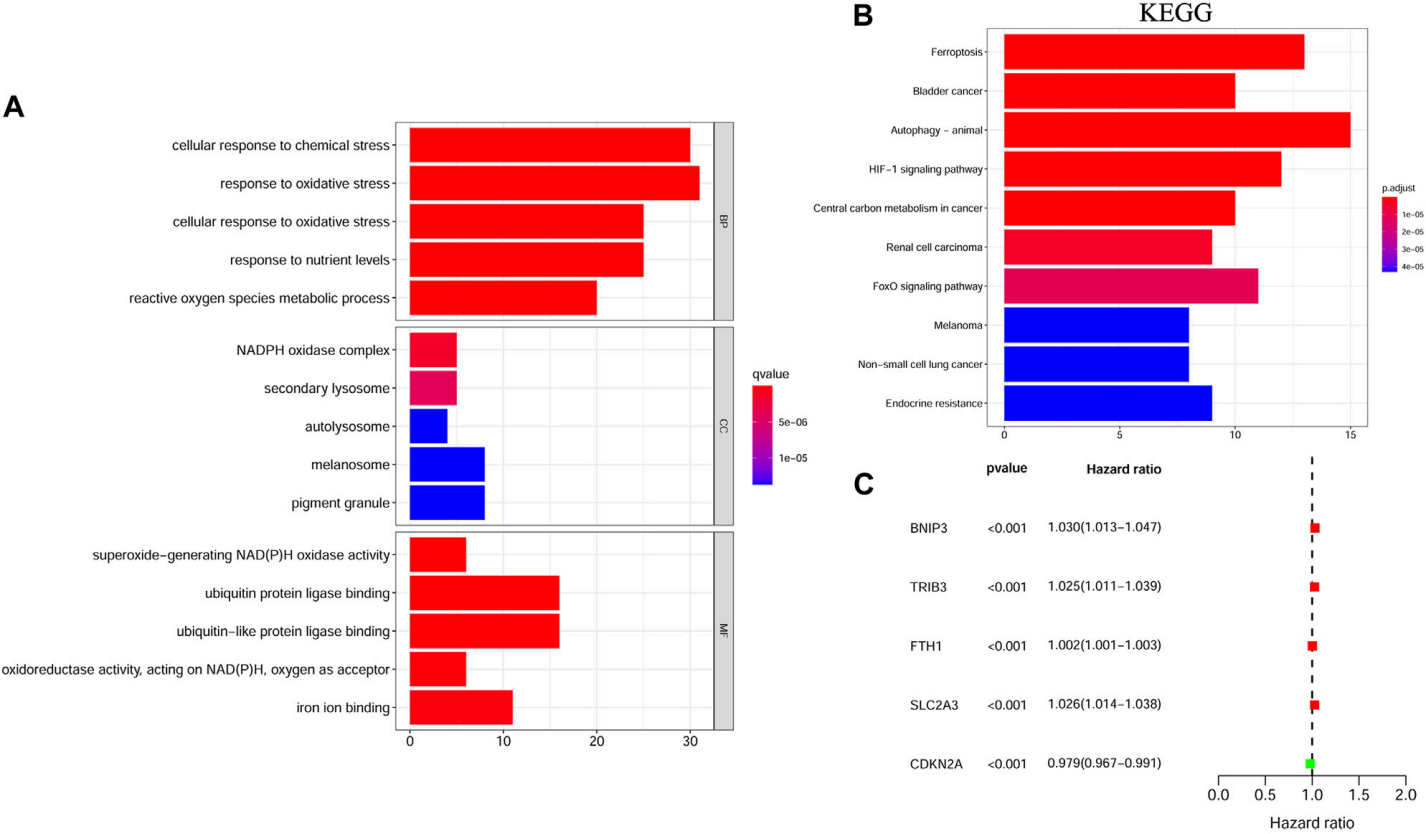
The functional enrichment analysis and univariate Cox regression analysis of ferroptosis-related DEGs between HNSCC samples and matched adjacent normal tissues. **(A)** GO term enrichment analysis of ferroptosis-related DEGs. **(B)** KEGG pathway enrichment analysis of ferroptosis-related DEGs. **(C)** A forest plot of the univariate Cox regression analysis with the prognostic FRGs. DEGs, Differentially expressed genes; HNSCC, Head and neck squamous cell carcinoma; GO, Gene ontology; KEGG, Kyoto Encyclopedia of Genes and Genomes; FRGs, Ferroptosis-related genes.

We filtered out 124 differentially expressed genes (DEGs) in HNSCC tissues vs. adjacent nontumorous tissues, 94 of which were upregulated and 30 were downregulated. As expected, GO and KEGG pathway enrichment analyses showed that these DEGs were mainly enriched in iron-related and metabolism-related molecular functions and ferroptosis-related and cancer-related pathways (Figures 1A,B). Moreover, univariate Cox regression analysis identified five differentially expressed FRGs related to OS of HNSCC, among which *FTH1*, *BNIP3*, *TRIB3*, and *SLC2A3* were high-risk FRGs (*p* < 0.001, hazard ratio [HR] > 1) and *CDKN2A* was low-risk FRGs (*p* < 0.001, hazard ratio [HR] < 1) (Figure 1C). We excluded *CDKN2A* from the following research considering that *CDKN2A* was overexpressed in HNSCC samples, but it seemed unreasonable that up-regulated *CDKN2A* was a favorable factor for the OS of HNSCC samples according to univariate Cox analysis.

### Construction of a Ferroptosis-Related Prognostic Model in the Training Cohort

Based on the four high-risk FRGs obtained above, we constructed a ferroptosis-related prognostic model via LASSO Cox regression analysis and calculated the regression coefficient of each model gene (Supplementary Table S1). The 4-gene signature was constructed on genes including *FTH1*, *BNIP3*, *TRIB3*, and *SLC2A3*. The risk score of each patient was calculated according to the expression and regression coefficient of model genes. Patients in the training cohort were then divided into a high-risk group (*n* = 243) and a low-risk group (*n* = 243) based on the median risk score. In the validation cohort, patients were also classified into high-risk (*n* = 165) and low-risk (*n* = 101) groups.

### External Validation of the Model Genes Using Online Database

**FIGURE 2:**
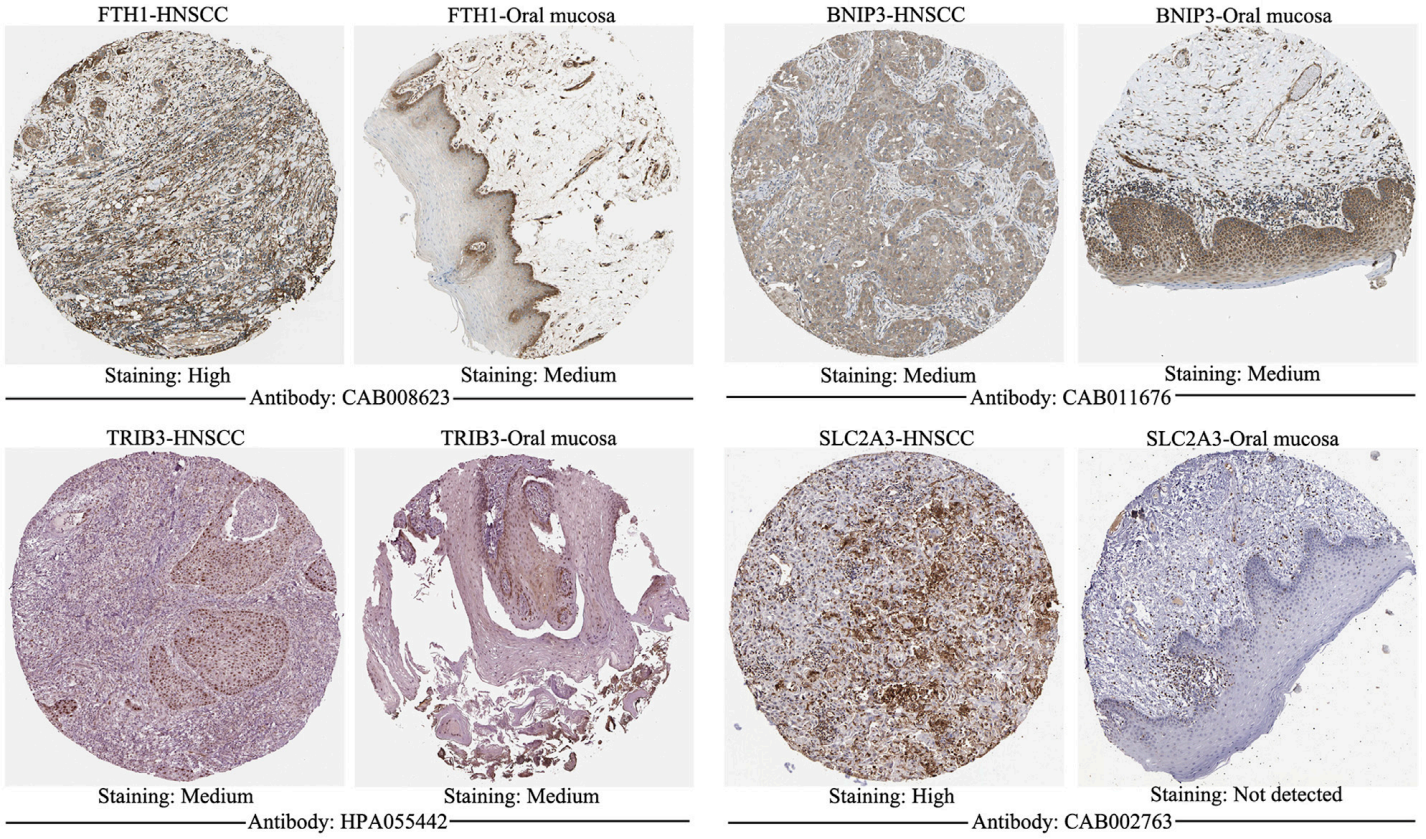
Immunohistochemistry of signature genes from the HPA. Representative images showing the expression of each gene in HNSCC tissues versus normal oral cavity mucosal tissues. HPA, Human Protein Atlas; HNSCC, Head and neck squamous cell carcinoma.

The signature genes were validated using mRNA expression and DNA copy number data from the Oncomine database. The levels of mRNA expression and DNA copy number of *FTH1*, *BNIP3*, *TRIB3*, and *SLC2A3* were all significantly elevated in HNSCC samples compared with those in normal samples (Table 2, Supplementary Figure S1), which were consistent with our results. Besides, the signature genes were also validated with IHC data from the HPA database (Figure 2).

**TABLE 2:** The DNA copy number and mRNA expression of the prognostic model genes between HNSCC and normal samples (ONCOMINE Database).

Model genes	Types	No. of patients	Types of HNSCC	*p*-value	t-value	FC	PMID/TCGA
FTH1	DNA copy number	290	HNSCC	1.33E-05	4.269	1.03	TCGA-HNSC
	DNA copy number	112	oral cavity SCC	9.88E-06	4.473	1.031	21853135
	mRNA expression	34	HNSCC	0.004	4.301	1.577	14676830
	mRNA expression	31	tongue SCC	3.20E-02	1.892	1.238	19138406
	mRNA expression	41	HNSCC	6.93E-07	6.423	2.07	14729608
	mRNA expression	57	oral cavity SCC	5.82E-10	7.371	1.543	21853135
	mRNA expression	15	tongue carcinoma	4.00E-04	3.673	2.819	17510386
	mRNA expression	31	NPC	0.022	2.205	1.366	16205657
	mRNA expression	31	tongue SCC	3.80E-02	1.803	1.276	15833835
	mRNA expression	16	oral cavity SCC	8.54E-06	6.858	2.973	15381369
BNIP3	DNA copy number	112	oral cavity SCC	0.006	2.541	1.01	21853135
	mRNA expression	34	HNSCC	0.032	2.132	1.347	14676830
	mRNA expression	15	tongue carcinoma	8.16E-04	3.532	2.006	17510386
	mRNA expression	26	tongue SCC	0.015	2.295	1.603	18254958
	mRNA expression	31	NPC	0.012	2.382	1.496	16912175
TRIB3	DNA copy number	112	oral cavity SCC	1.15E-08	5.989	1.054	21853135
	DNA copy number	290	HNSCC	6.01E-05	3.891	1.037	TCGA-HNSC
	mRNA expression	26	tongue SCC	4.40E-04	3.762	1.88	18,254,958
	mRNA expression	16	oral cavity SCC	0.005	2.901	1.275	15381369
	mRNA expression	15	tongue carcinoma	0.017	2.26	1.309	17510386
	mRNA expression	31	NPC	0.011	2.439	1.302	16912175
	mRNA expression	41	HNSCC	0.033	1.907	1.198	14729608
	mRNA expression	57	oral cavity SCC	0.031	1.923	1.176	21853135
SLC2A3	DNA copy number	290	HNSCC	5.83E-07	4.935	1.065	TCGA-HNSC
	DNA copy number	112	oral cavity SCC	5.19E-04	3.363	1.031	21853135
	mRNA expression	34	HNSCC	9.57E-17	17.13	5.59	14676830
	mRNA expression	41	HNSCC	2.39E-23	17.315	16.709	14729608
	mRNA expression	57	oral cavity SCC	1.36E-06	5.156	1.65	21853135
	mRNA expression	26	tongue SCC	0.004	2.774	1.115	18254958

FC, fold change; NPC, nasopharyngeal carcinoma; SCC, squamous cell carcinoma; HNSCC, head and neck squamous cell carcinoma; TCGA, the Cancer Genome Atlas.

### Prognostic Values of the Ferroptosis-Related Signature in Training and Validation Cohorts

**FIGURE 3:**
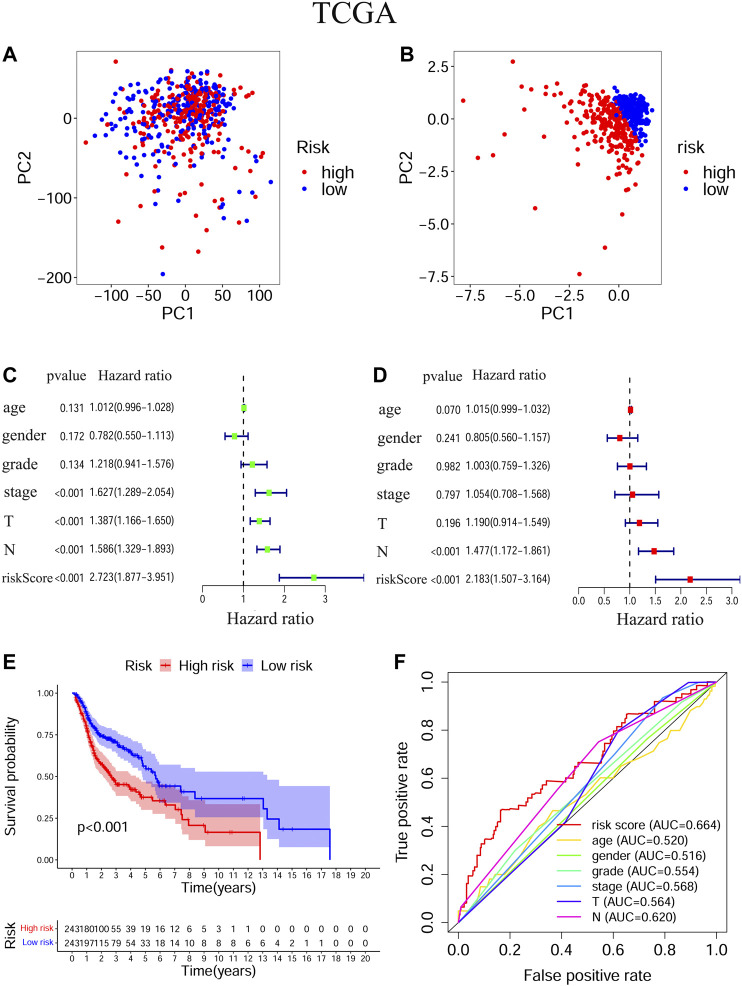
Evaluation of the ability of the ferroptosis-related signature to predicting prognosis in HNSCC patients based on TCGA-HNSCC cohort. **(A)** PCA based on all ferroptosis-related genes. **(B)** PCA based on ferroptosis-related signature to distinguish tumors from normal samples. **(C)** Univariate Cox regression analysis of the signature and clinical features. **(D)** Multivariate Cox regression analysis of the signature and clinical features. **(E)** Kaplan–Meier survival curves of the prognostic signature. **(F)** The 1-year ROC curves and AUC values of the signature and clinical features. PCA, Principal component analysis, HNSCC, Head and neck squamous cell carcinoma; TCGA, The Cancer Genome Atlas; ROC, Receiver operating characteristic; AUC, Area under curve.

**FIGURE 4:**
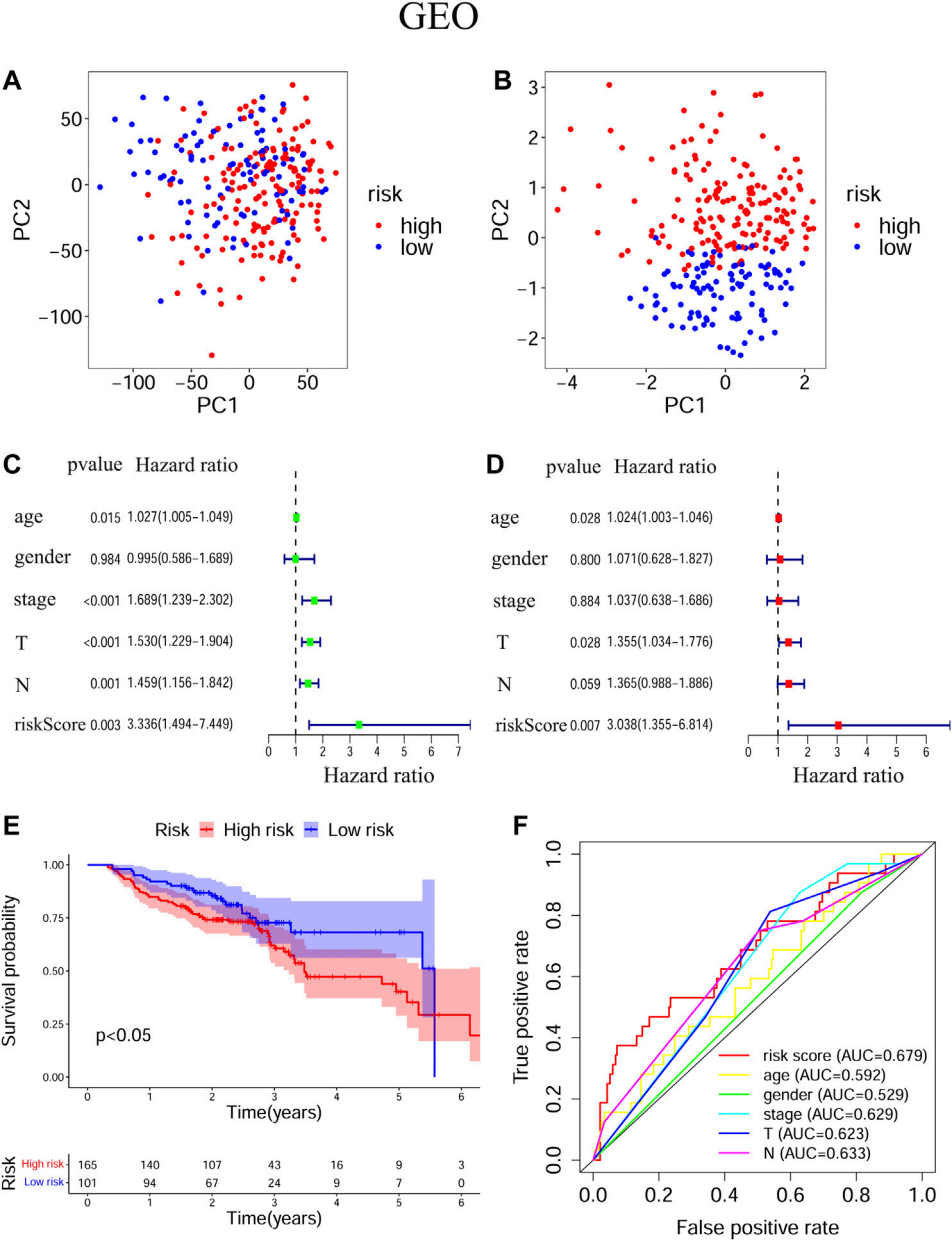
Evaluation of the ability of the ferroptosis-related signature to predicting prognosis in HNSCC patients based on GEO-HNSCC cohort. **(A)** PCA based on all ferroptosis-related genes. **(B)** PCA based on ferroptosis-related signature to distinguish tumors from normal samples. **(C)** Univariate Cox regression analysis of the signature and clinical features. **(D)** Multivariate Cox regression analysis of the signature and clinical features. **(E)** Kaplan–Meier survival curves of the prognostic signature. **(F)** The 1-year ROC curves and AUC values of the signature and clinical features. PCA, Principal component analysis; HNSCC, Head and neck squamous cell carcinoma; GEO, The Gene Expression Omnibus; ROC, Receiver operating characteristic; AUC, Area under curve.

The transcriptional levels of *FTH1*, *BNIP3*, *TRIB3*, and *SLC2A3* were all significantly upregulated in the high-risk group compared to the low-risk group according to both training and validation cohorts (Supplementary Figure S2). Based on TCGA-HNSCC (Figures 3A,B) and GEO-HNSCC (Figures 4A,B) datasets, the PCA before and after establishment of the prognostic risk model indicated that patients in different risk subgroups were distributed into two directions well. The Kaplan–Meier survival curves showed the patients with HNSCC in the low-risk group had a significantly better OS than those in the high-risk group (TCGA-HNSCC cohort: Figure 3E, *p* < 0.001; GEO-HNSCC cohort: Figure 4E, *p* < 0.05). In the TCGA-HNSCC cohort, the 5-years survival rate of the high-risk group was 0.375 (95% CI: 0.300–0.469), while that of the low-risk group was 0.563 (95% CI: 0.477–0.663) (Figure 3E). Meanwhile, the 5-years survival rate of the high-risk group was 0.402 (95% CI: 0.290–0.559), whereas that of the low-risk group was 0.682 (95% CI: 0.562–0.829) (Figure 4E) in the GEO-HNSCC cohort. Apparently, patients in high-risk group had a lower 5-years survival rate than those in low-risk group. Univariate Cox regression analysis of OS indicated that several clinical characteristics, including clinical stage (*p* < 0.001), T stage (*p* < 0.001), and N stage (*p* < 0.001), as well as the risk score (TCGA-HNSCC: *p* < 0.001; GEO-HNSCC: *p* < 0.01), were effective prognostic indicators for patients with HNSCC (3C, 4C). Moreover, multivariate Cox regression analysis of OS demonstrated that the risk score was an independent prognostic predictor for HNSCC patients (TCGA-HNSCC: *p* < 0.001; GEO-HNSCC: *p* < 0.01) (3D, 4D). In order to explore the sensitivity and specificity of clinical characteristics and the risk signature with regard to survival prediction, we drew ROC curves and then calculated the AUC values (3F, 4F; Supplementary Figures S3A, S3B). The AUC values at 1 year in training and validation cohorts were 0.664 and 0.679, respectively, and the prognostic accuracy of this signature was higher than that of all six clinical characteristics (3F, 4F). Considering that HNSCC contains multiple tumors at different anatomical sites, we analyzed the correlation between anatomical sites and the risk score using Kruskal test (Supplementary Figures S4E) according to HNSCC-TCGA cohort. The risk score was shown to be significantly associated with different anatomical sites (*p <* 0.05).

### A Predictive Nomogram Based on TCGA-HNSCC Cohort

**FIGURE 5:**
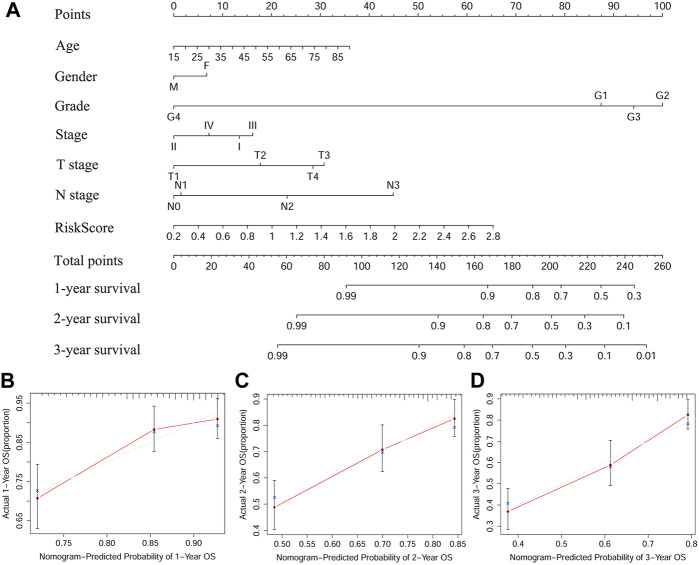
A predictive nomogram based on the signature. **(A)** The nomogram for predicting 1, 2, and 3-years OS of HSNCC with clinical indicators and risk score. **(B–D)** The 1, 2, and 3-years calibration curves of TCGA-HNSCC. X axis represents predicted survival time and Y axis indicates actual survival time. HNSCC, Head and neck squamous cell carcinoma, TCGA, The Cancer Genome Atlas; OS, Overall survival.

The nomogram is an effective method to predict the onset, progression or prognosis of diseases by integrating multiple risk factors. We successfully constructed a nomogram based on seven risk factors, including age, gender, grade, stage, T stage, N stage, and the ferroptosis-related signature, to predict 1, 2, and 3-years OS in TCGA-HNSC cohort (Figure 5A). Each risk factor had its own point and all contributed to the total point of each patient, according to which we got to know the 1, 2, and 3-years OS probabilities of patients. Calibration curves indicated that the predicted 1, 2, and 3-years OS probabilities were all well consistent with the actual ones (Figures 5B–D).

### Functional Enrichment Analysis of Different Risk Groups

**FIGURE 6:**
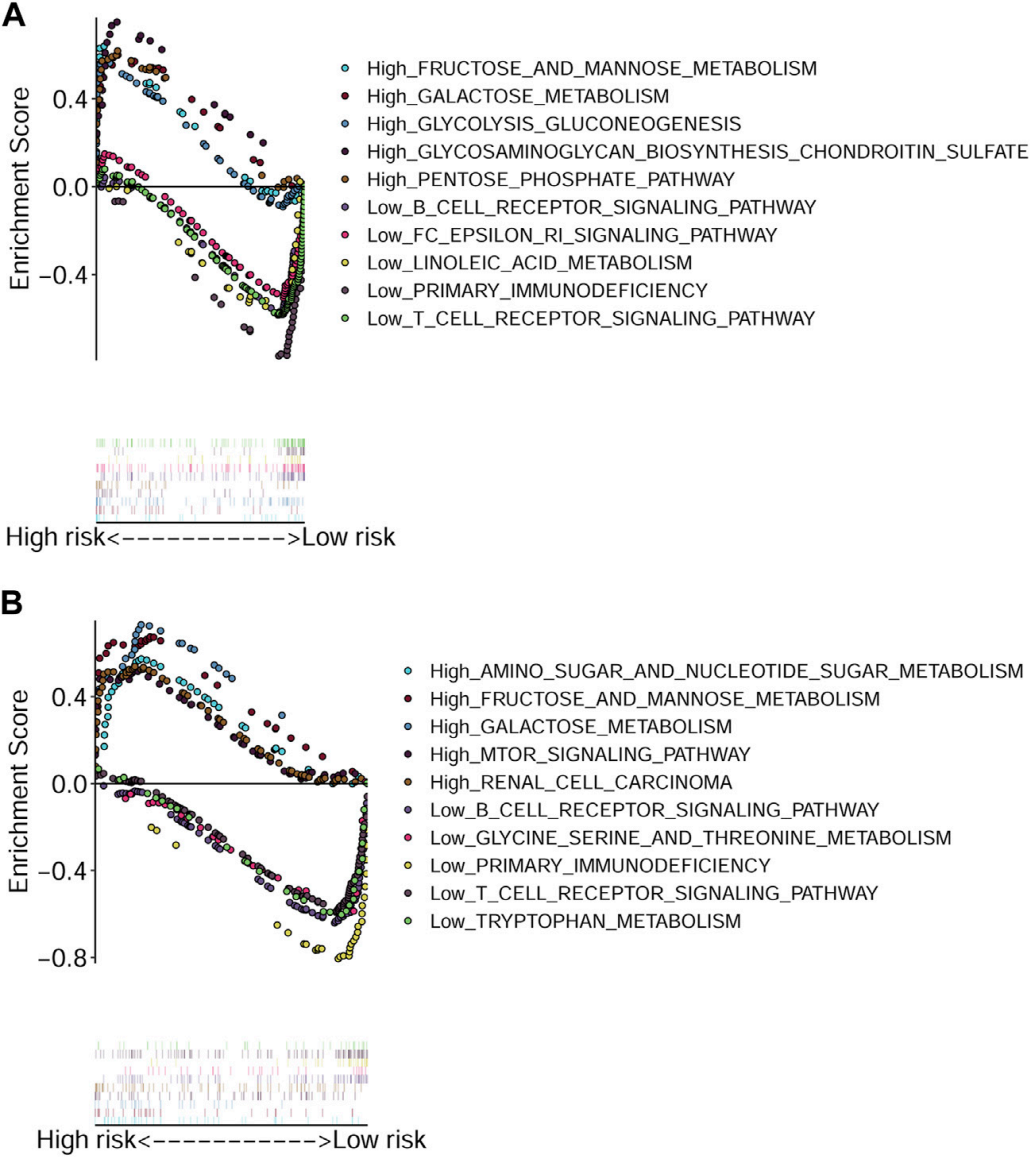
Gene Set Enrichment Analysis based on the signature. **(A)** Enriched pathways in the high and low-risk groups in TCGA cohort. **(B)** Enriched pathways in the high and low-risk groups in GEO cohort. GEO, The Gene Expression Omnibus; TCGA, The Cancer Genome Atlas.

The GSEA was performed to explore the active pathways or functions enriched in the low and high-risk groups according to TCGA-HNSCC cohort (Figure 6A) and GEO-HNSCC cohort (Figure 6B). Results of GSEA in the training and validation cohorts were basically the same and the detailed information was shown in Figures 6A,B. Briefly speaking, pathways enriched in the high-risk group were mainly energy metabolism-related, while among the low-risk group, the most enriched pathways were closely related to immunity.

### Immune Correlation of the Constructed Model

**FIGURE 7:**
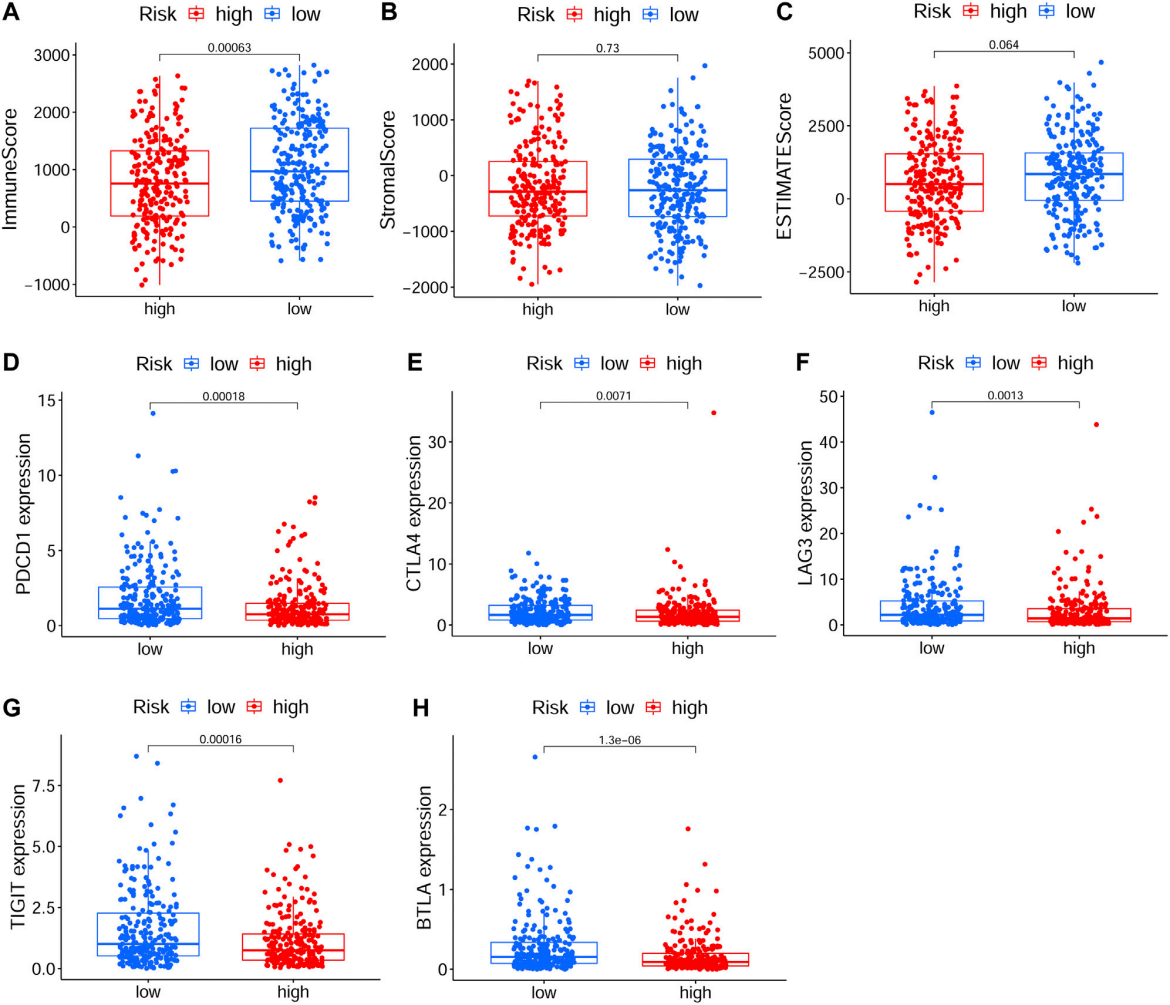
The analyses of immune microenvironment and immune checkpoints of the prognostic signature based on TCGA cohort. **(A–C)** The immune, stromal, and ESTIMATE scores in different risk groups. **(D–H)** The expression levels of immune checkpoints, including *PD-1*(*PDCD1*), *CTLA4*, *LAG3*, *TIGIT*, and *BTLA*, in different risk groups. The *p* values were showed as: *p < 0.05; **p < 0.01; ***p < 0.001. TCGA, The Cancer Genome Atlas.

**FIGURE 8:**
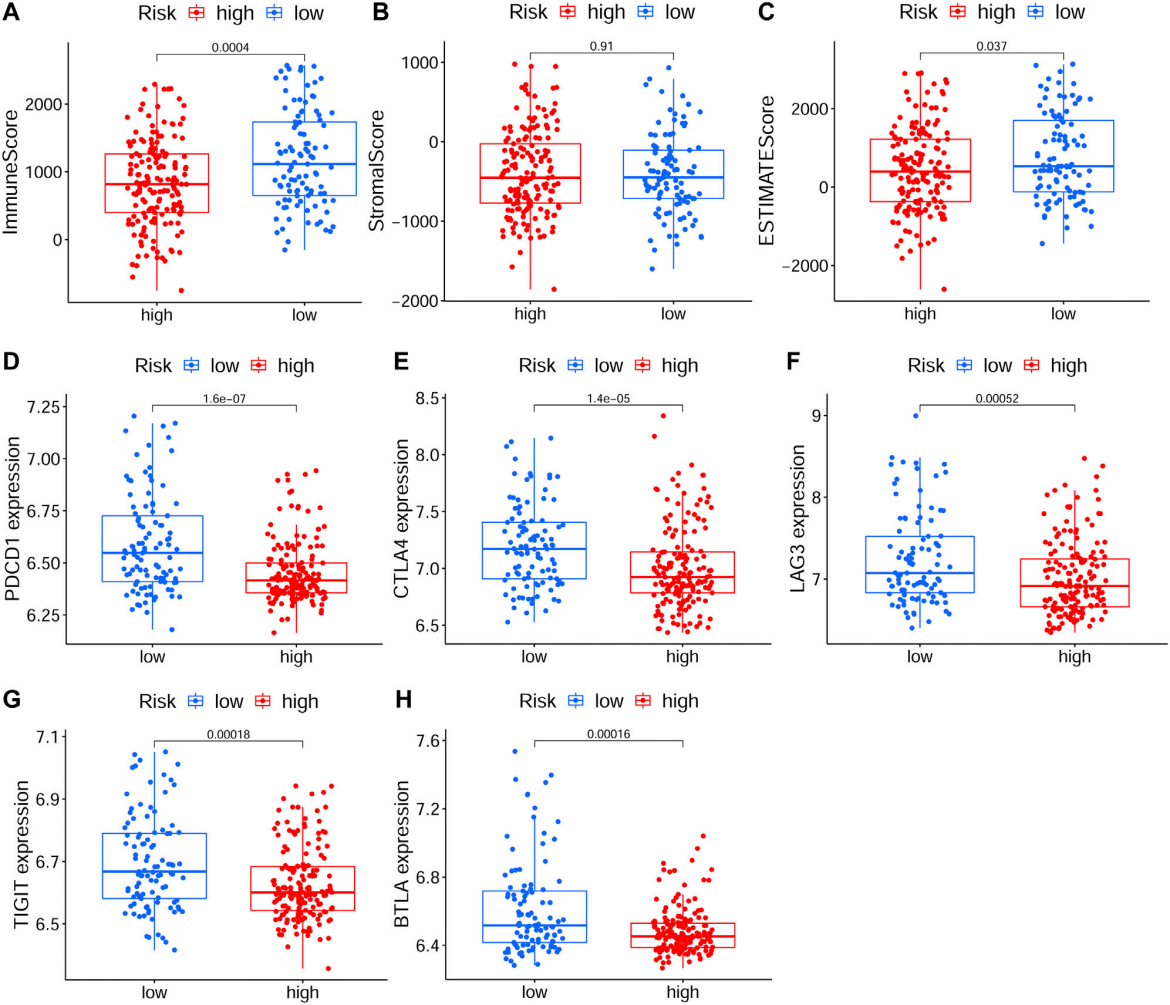
The analyses of immune microenvironment and immune checkpoints of the prognostic signature based on GEO cohort. **(A–C)** The immune, stromal, and ESTIMATE scores in different risk groups. **(D–H)** The expression levels of immune checkpoints, including *PD-1*(*PDCD1*), *CTLA4*, *LAG3*, *TIGIT*, and *BTLA*, in different risk groups. The *p* values were showed as: *p < 0.05; **p < 0.01; ***p < 0.001. GEO, The Gene Expression Omnibus.

**FIGURE 9:**
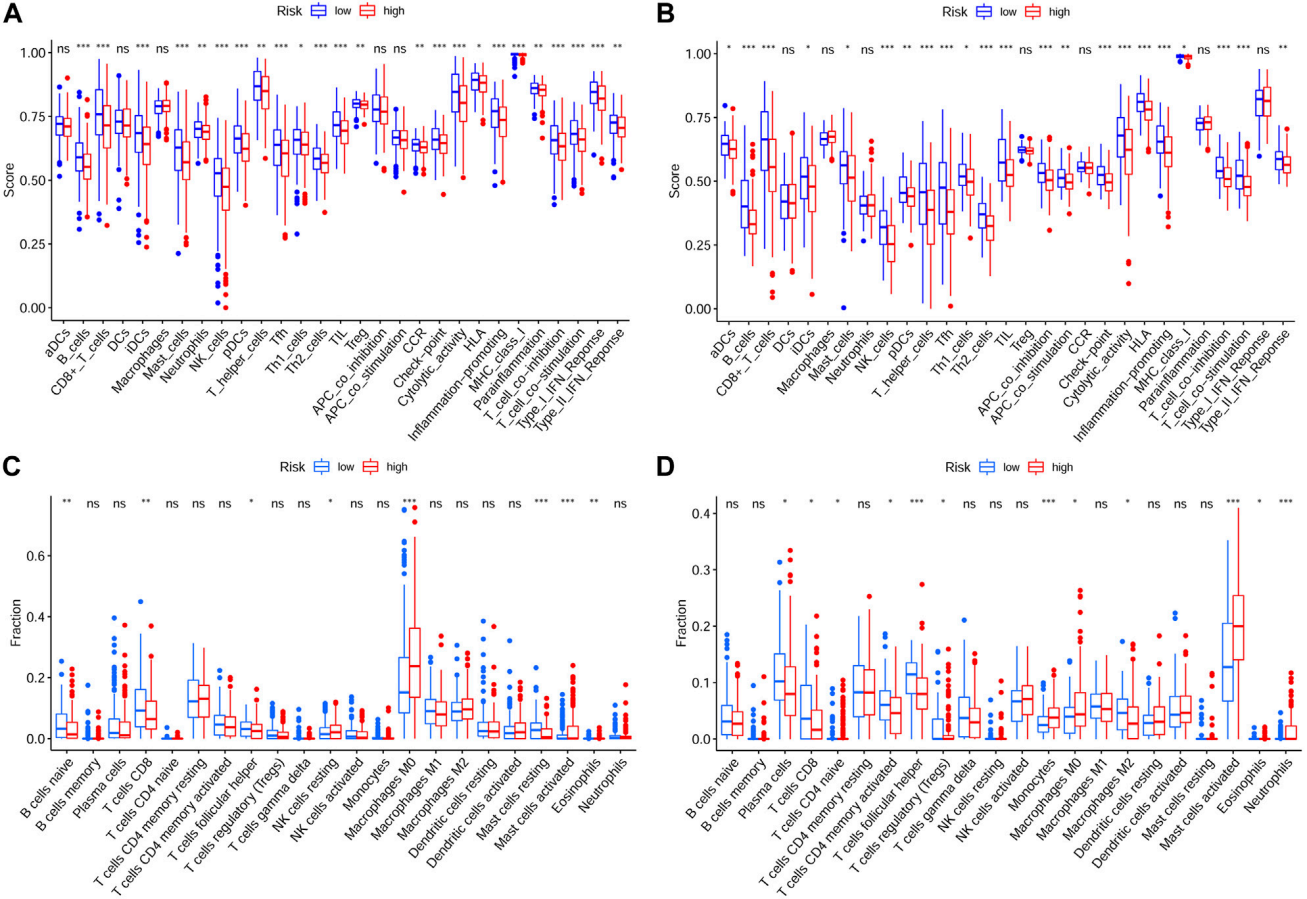
Analysis of immune cells and immune functions of the prognostic signature. Analysis of infiltrating scores of 16 immune cells and activity of 13 immune-related pathways in different risk groups via ssGSEA based on TCGA cohort **(A)** and GEO cohort **(B)**. Analysis of the abundance of 22 immune cells via CIBERSORT algorithm based on TCGA cohort **(C)** and GEO cohort **(D)**. The *p* values were showed as: *p < 0.05; **p < 0.01; ***p < 0.001. ssGSEA, single-sample Gene Set Enrichment Analysis; GEO, The Gene Expression Omnibus; TCGA, The Cancer Genome Atlas.

To explore relationship between the prognostic risk model and immune status, the stromal, immune and ESTIMATE scores, immune cells, immune-related functions/pathways, and immune checkpoints were estimated in different risk groups. According to TCGA-HNSCC and GEO-HNSCC cohorts, the immune scores in low-risk group were remarkably higher than that in high-risk group (*p* < 0.001, 7A, 8A). Among GEO-HNSCC cohort, patients in low-risk group had higher ESTIMATE scores (*p* < 0.05, Figure 8C), indicating a higher level of tumor purity. Although there was no significant difference in ESTIMATE scores between the two risk groups in TCGA-HNSCC cohort (*p* = 0.064, Figure 7C), the low-risk group still had a higher mean value of ESTIMATE scores than the high-risk group. Next, the ssGSEA and CIBERSORT algorithm were used to evaluate the enrichment scores of immune cells and immune-related functions in different risk groups. We found that the infiltration levels of B cells, CD8^+^ T cells, mast cells, NK cells, iDCs, pDCs, helper T cells, follicular helper T cells, Tfh, Th1 cells, Th2 cells, TILs, M0 macrophage cells, eosinophils and activated mast cells were significantly different between low and high-risk groups in both TCGA-HNSCC (Figures 9A,C) and GEO-HNSCC cohorts (Figures 9B,D). Interestingly, the low-risk group was found to have a higher infiltration level of regulatory T cell (Tregs) as shown in Figures 9A,D, which might appear contradictory to the immunosuppressive nature of Tregs. Furthermore, compared with the high-risk group, the scores of check-point, cytolytic activity, HLA, MHC class I, T cell co-stimulation, T cell co-inhibition, and type II IFN response were all elevated in the low-risk group in both the training and validation cohorts (Figures 9A,B). To further evaluate the availability of the constructed risk model in immunotherapy, we explored immune checkpoints of HNSCC (Kok, 2020) in different risk groups and discovered that the expression levels of *PD-1*(*PDCD1*), *CTLA4*, *LAG3*, *TIGIT*, and *BTLA* were all significantly upregulated in the low-risk group compared with the high-risk group according to TCGA-HNSCC and GSE65858 cohorts (all *p* < 0.01, Figures 7D–H, 8D–H).

### Clinical Values of the Prognostic Model and Genes in Chemotherapy

**FIGURE 10:**
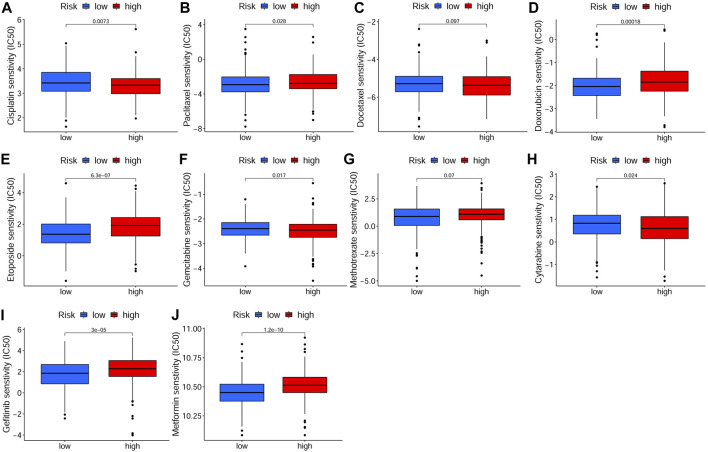
Drug sensitivity analysis in TCGA cohort. The IC50 of chemotherapeutic drugs of HSNCC, including Cisplatin **(A)**, Paclitaxel **(B)**, Docetaxel **(C)**, Doxorubicin **(D)**, Etoposide **(E)**, Gemcitabine **(F)**, Methotrexate **(G)**, Cytarabine **(H)**, Gefitinib **(I)** and Metformin **(J)**, in different risk groups. There were 243 samples in both low- and high-risk groups. TCGA: The Cancer Genome Atlas, IC50: Half-maximal inhibitory concentration.

**FIGURE 11:**
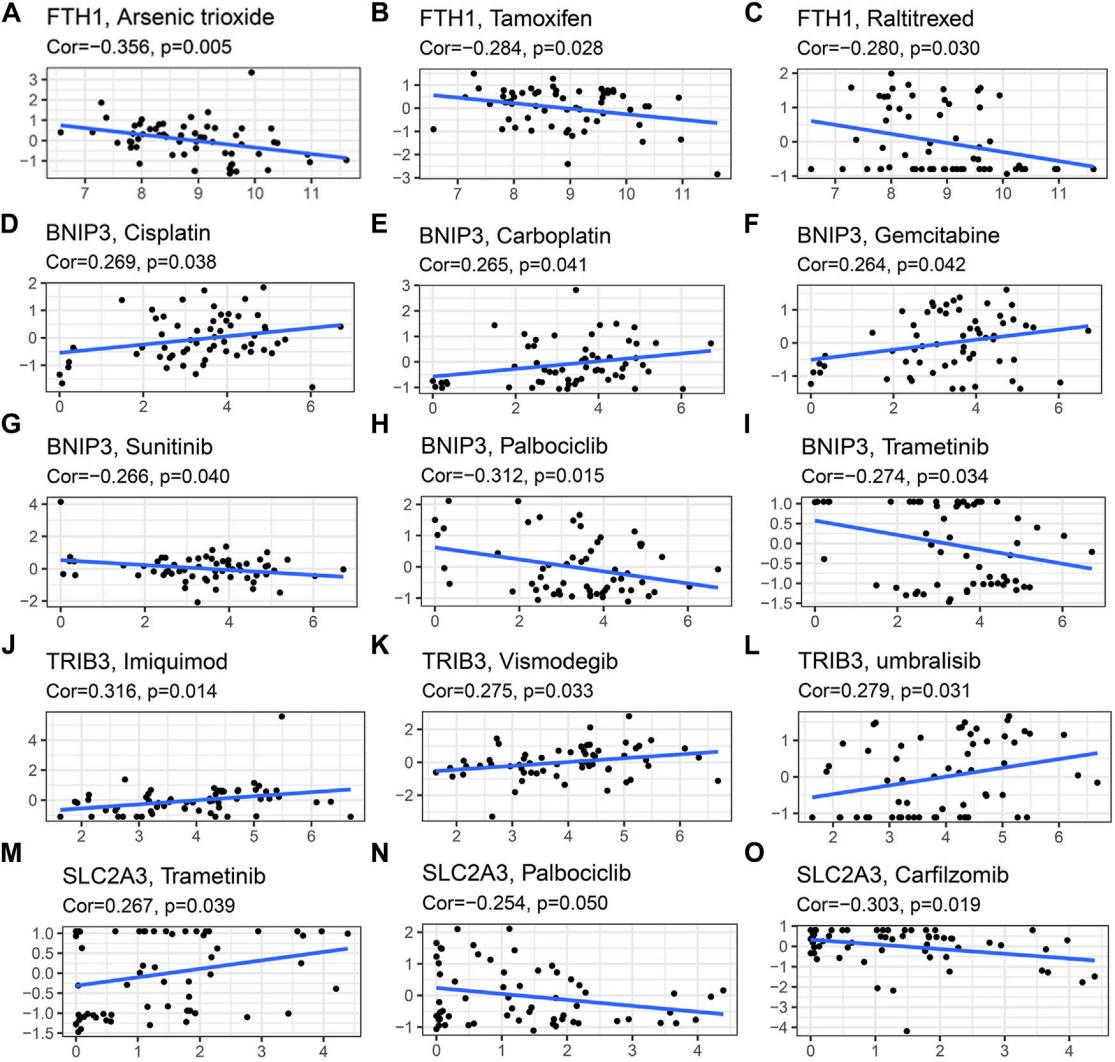
Scatter plots of relationship between expression of model genes and drug sensitivity of cancer cells to some FDA approved drugs. **(A–C)** *FTH1* **(D–I)** *BNIP3* **(J–L)** *TRIB3* **(M–O)** *SLC2A3*.

In order to investigate the correlation of the prognostic signature with efficacy of chemotherapy in HNSCC, we used IC50 to predict the treatment response to common chemotherapeutic drugs in TCGA cohort. We discovered that the IC50 of Cisplatin (*p* < 0.01, Figure 10A), Gemcitabine (*p* < 0.05, Figure 10F) and Cytarabine (*p* < 0.05, Figure 10H) was significantly higher in the low-risk group, whereas, the high-risk group had a higher IC50 of Paclitaxel (*p* < 0.05, Figure 10B), Doxorubicin (*p* < 0.001, Figure 10D) and Etoposide (*p* < 0.001, Figure 10E), which indicated that the risk signature could be an indicator for predicting sensitivity of chemotherapeutic drugs. However, there was no significant difference in the IC50 of Docetaxel (Figure 10C) and methotrexate (Figure 10G). In addition, the high-risk group was more sensitive to some novel anti-cancer drugs, such as Gefitinib (*p* < 0.001, Figure 10I) and Metformin (*p* < 0.001, Figure 10J). Based on the CellMiner database, we next explored the correlation between expression of signature genes and the resistance/sensitivity of pan-cancer cells to chemotherapeutic drugs. As a result, all signature genes were significantly associated with sensitivity of some chemotherapeutic drugs (*p* < 0.05, Supplementary Table S2). For example, the expression of *FTH1* had a positive correlation with drug resistance of cancer cells to Arsenic trioxide, Tamoxifen and Raltitrexed (Figures 11A–C). Increased expression of *BNIP3* was correlated with increased drug sensitivity of cancer cells to Cisplatin, Carboplatin and Gemcitabine (Figures 11D–F), whereas it was negatively correlated with drug sensitivity of cancer cells to Sunitinib, Palbociclib, and Trametinib (Figures 11G–I). Upregulated *TRIB3* was associated with increased drug sensitivity of cancer cells to Imiquimod, Vismodegib and umbralisib (Figures 11J–L). As for risk gene *SLC2A3*, the expression had a positive correlation with drug sensitivity of cancer cells to Trametinib, but showed a negative correlation with drug sensitivity of cancer cells to Palbociclib and Carfilzomib (Figures 11M–O).

## Discussion

As a process of cell death, ferroptosis plays a crucial role in tumorigenesis and has the ability to strongly inhibit tumor growth (Lu et al., 2017). Previous studies have indicated that ferroptosis can enhance the sensitivity of chemotherapeutic drugs (Lu et al., 2017; El Hout et al., 2018), and therefore, the induction of ferroptosis may provide a new therapeutic approach for cancer, especially drug-resistant tumors. As it has been shown that inhibition of FRGs can overcome cisplatin resistance in HNSCC via the induction of ferroptosis (Roh et al., 2017; Kim et al., 2018; Lee et al., 2020), we explored the mRNA expression levels of 187 retrieved FRGs in HNSCC and built a novel prognostic model based on these genes.

In this study, we constructed a prognostic model for HSNCC patients based on four FRGs (*FTH1*, *BNIP3*, *TRIB3*, and *SLC2A3*), which were all high-risk genes. FTH1 is a vital iron regulatory protein and an inhibitor of ferroptosis by binding Fe^2+^ (Song et al., 2016). The upregulation of *FTH1* is correlated with cervical lymph node metastasis and poor prognosis of patients with HNSCC (Hu et al., 2019). *SLC2A3* encodes the glucose transporter 3 (GLUT3), which may inhibit ferroptosis (Jiang et al., 2017), has a tumorigenic role in many malignancies and could be a promising target for anticancer therapy (Masin et al., 2014; Dai et al., 2020). Moreover, high expression of GLUT3 is remarkably associated with poor prognosis in oral squamous cell carcinoma, probably resulting from the enhanced glycolytic metabolism of more aggressive cancer cells (Ayala et al., 2010). The above findings are consistent with our results and indicate the reliability of our prognostic signature. The expression levels of *BNIP3* and *TRIB3* are both upregulated during ferroptosis induced by erastin or RSL3, which indicates they may promote ferroptosis (Dixon et al., 2014; Yang et al., 2014). Meanwhile, although both *BNIP3* and *TRIB3* are found to have a strong impact on the development, progression, and prognosis of multiple cancers (Zhang and Ney, 2009; Gorbunova et al., 2020; Stefanovska et al., 2021), their roles in HNSCC are still inconclusive.

Univariate Cox regression analysis indicated that these four FRGs were significantly associated with the OS of HNSCC. Based on Kaplan-Meier analysis, the higher risk score was significantly correlated with a poorer prognosis in patients with HNSCC. Meanwhile, our signature was found to be an independent prognostic indicator according to multivariate Cox regression analysis of OS. The AUC values of the risk score in both training and validation cohorts were all higher than those of the six clinical features, demonstrating the accuracy of our signature as a prognostic marker. Among HNSCCs, tumors localized at oropharynx presented the best survival probabilities, followed by the nasopharynx, oral cavity, larynx, and hypopharynx (Gootee et al., 2020) and in our study, tumors at oropharynx demonstrated the lowest risk score, followed by the oral cavity, larynx, and hypopharynx, which indicated that the constructed model should be an effective tool for predicting the prognosis of HNSCC patients. In addition, the model genes were all validated in HNSCC using mRNA expression and DNA copy number data from the Oncomine database and IHC data from the HPA database. Then, we established a nomogram based on the ferroptosis-related signature and six clinical indicators. Calibration curves showed our nomogram could accurately predict 1, 2, and 3-years survival probabilities for HNSCC patients, which illustrated it was a good predictor of survival in HNSCC patients with short-term follow-ups.

We next explored the involved pathways and functions in different risk groups using GSEA in both TCGA-HNSCC and GEO-HNSCC cohorts. Results of the two independent datasets were consistent, which both indicated that pathways activated in the high-risk group were mainly energy metabolism-related signaling pathways. Energy metabolism was reported to be a regulator of ferroptosis (Ma et al., 2020) and meanwhile, it was also known to be able to increase the survival and proliferative capacity of cancer cells, even under nutrient-limiting conditions (Li and Zhang, 2016), which was consistent with the poor prognosis of high-risk patients. However, active pathways enriched in the low-risk group were mainly immune-related functions. Wang et al. reported that beyond traditional mechanisms, CD8^+^ T cells could also suppress tumor growth by inducing ferroptosis, which was the first direct evidence of the connection between ferroptosis and antitumor immunity (Wang et al., 2019). The induction of ferroptosis can enhance the antitumor activity of immune checkpoint inhibitors (ICIs), even in ICI-resistant tumors (Tang et al., 2020). In addition, immunotherapy, especially ICI treatment, has been proven to be an effective and promising treatment for recurrent or metastatic HNSCC patients (Cohen et al., 2019). Therefore, the combination of ICIs and ferroptosis inducers may provide potential therapeutic strategies for intractable HNSCC patients.

To further explore the correlation between the ferroptosis-related signature and immune status of HNSCC patients, we evaluated the immune and ESTIMATE scores, immune infiltration cells, immune-related functions, and immune checkpoints in different risk groups. The immune score has been considered as a new approach for defining cancer classification and also a novel prognostic indicator for multiple cancers (Galon et al., 2012). High immune score indicated a good prognosis (Galon et al., 2012; Tahkola et al., 2018) and, in our study, the immune score in low-risk group was obviously higher according to both TCGA-HNSCC and GEO-HNSCC datasets. Previous studies revealed that CD8^+^ T cells (Mandal et al., 2016; Spector et al., 2019), mast cells (Cheng et al., 2021), NK cells (Bisheshar et al., 2020), TILs (Spector et al., 2019), and CD4^+^ follicular helper T cells (Cillo et al., 2020) have been shown to have a positive role in antitumor immunity and prognosis of HNSCC. Based on the training and validation cohorts, the infiltration levels of CD8^+^ T cells, mast cells, NK cells, TILs, and follicular helper T cells were all significantly higher in low-risk group. The higher infiltration level of Tregs in low-risk group may appear contradictory to their immunosuppressive nature, whereas the tumor infiltration by FOXP3^+^CD4^+^ Tregs is found to be positively correlated with better locoregional control of the head and neck cancer (Badoual et al., 2006) and the high Tregs fraction has a positive correlation with good prognosis in HNSCC (Cillo et al., 2020). The infiltration level of activated mast cells was significantly higher in high-risk group. Activated mast cells can induce epithelial-to-mesenchymal transition and thus promote tumor progression (Visciano et al., 2015), which may explain the poor survival in high-risk group. Meanwhile, the Cytolytic activity is correlated with improved prognosis and counter-regulatory activities, which limit the immune response in cancers (Rooney et al., 2015). Type II IFN performs a vital role in tumor immune surveillance, stimulating antitumor immunity and promoting tumor recognition and elimination, and as expected, type II IFN response is associated with the prognosis in some cancers (Castro et al., 2018). However, the cytolytic activity and type II IFN response in the high-risk group notably reduced, which may make a contribution to its poor prognosis. Besides, compared with the high-risk group, the low-risk group presented significantly upregulated expression levels of immune checkpoint molecules, including *PD-1*, *CTLA4*, *LAG3*, *TIGIT*, and *BTLA*, which indicated patients in the low-risk group could be more suitable for ICI treatment. In conclusion, the above results of immunity analyses cohere with the results of our functional enrichment analysis using GSEA, may elucidate the immune mechanism by which the ferroptosis-related signature influences prognosis of HNSCC patients and can help clinicians to perform personalized immunotherapy.

In addition to immunotherapy, we also explored the efficacy of some common chemotherapy drugs in different risk groups. Miyazawa et al. found that Cisplatin inhibited the iron regulatory protein 2, caused intracellular iron deficiency and thus leaded to dysregulated iron metabolism, which could finally result in cancer cell death (Miyazawa et al., 2019). Moreover, Cisplatin was reported to be an inducer for ferroptosis and combination therapy of Cisplatin and erastin presented significant synergistic effect on their anti-tumor activity (Guo et al., 2018). Doxorubicin was found to increase mitochondrial iron levels, leading to mitochondrial iron accumulation (Ichikawa et al., 2014) and meanwhile, correction of iron metabolism abnormalities could enhance sensitivity of cancer cells to Doxorubicin (Chekhun et al., 2013). Accordingly, multiple chemotherapy drugs have close relationships with iron metabolism and ferroptosis, which implies the possible values of ferroptosis-related signature for selecting optimal chemotherapy drugs. Based on the estimated IC50, patients in low-risk group showed more sensitive response to Cisplatin, Gemcitabine and Cytarabine, whereas patients in high-risk group were more sensitive to Paclitaxel, Doxorubicin and Etoposide, which indicated that the risk signature could contribute to the selection of optimal chemotherapy strategy. Tang et al. (2019) reported that although Gefitinib could not prolong the survival time for HNSCC patients, it could improve the quality of life for recurrent patients. Gulati et al. (2020) demonstrated that combining metformin with chemoradiotherapy could improve the rates of OS and progression-free survival (PFS) in patients with locally advanced HNSCC. And thus, as novel anti-cancer drugs, Gefitinib, and Metformin may be promising drugs for patients with recurrent or advanced HNSCC. Based on the risk signature, patients in high-risk group showed more sensitive response to Gefitinib and Metformin, which conformed with the previous studies. Meanwhile, on the basis of CellMiner database, we discovered that the expression of some model genes was positively correlated with drug resistance/sensitivity of a few drugs approved by FDA. For instance, elevated expression of *BNIP3* showed a correlation with drug sensitivity of cancer cells to Cisplatin and Gemcitabine. Considering their contributions to the PFS of patients with recurrent or metastatic nasopharyngeal carcinoma, Gemcitabine plus Cisplatin has been established as the standard first-line treatment option for these patients (Zhang et al., 2016). Ferroptosis induction is considered as a promising approach to overcome drug resistance via targeting cancer stem cells (CSCs) (Elgendy et al., 2020) and previous studies showed that the inhibition of some FRGs could reverse chemotherapy resistance of patients with HNSCC (Roh et al., 2017; Kim et al., 2018; Lee et al., 2020). Hence, targeting prognostic FRGs associated with drug resistance/sensitivity may be a promising therapeutic strategy for patients with drug resistance or probably can aid in drug sensitivity.

In our study, we constructed a novel prognostic signature of four FRGs for patients with HNSCC. According to TCGA-HNSCC cohort and GEO-HNSCC cohort, this signature was proven to be an independent prognostic indicator with significant prognostic value for HNSCC. Besides, the ferroptosis-related signature may be able to help clinicians identify the patients who may be suitable for immunotherapy and choose appropriate chemotherapy drugs for HNSCC patients. In brief, our findings provide additional information on the interactions between FRGs and the prognosis, immune status and chemotherapy sensitivity of HNSCC, which may contribute to the development of personalized chemotherapy or immunotherapy strategies and the identification of novel treatment targets for HNSCC.

## Data Availability

The original contributions presented in the study are included in the article/Supplementary Material, further inquiries can be directed to the corresponding author.
