# Supplementary figures and images for: A Ferroptosis-Related Gene Signature for Predicting the Prognosis and Drug Sensitivity of Head and Neck Squamous Cell Carcinoma
Source: Front Genet. 2021 Oct 21;12:755486. doi: 10.3389/fgene.2021.755486 (PMC8566369; doi:10.3389/fgene.2021.755486)

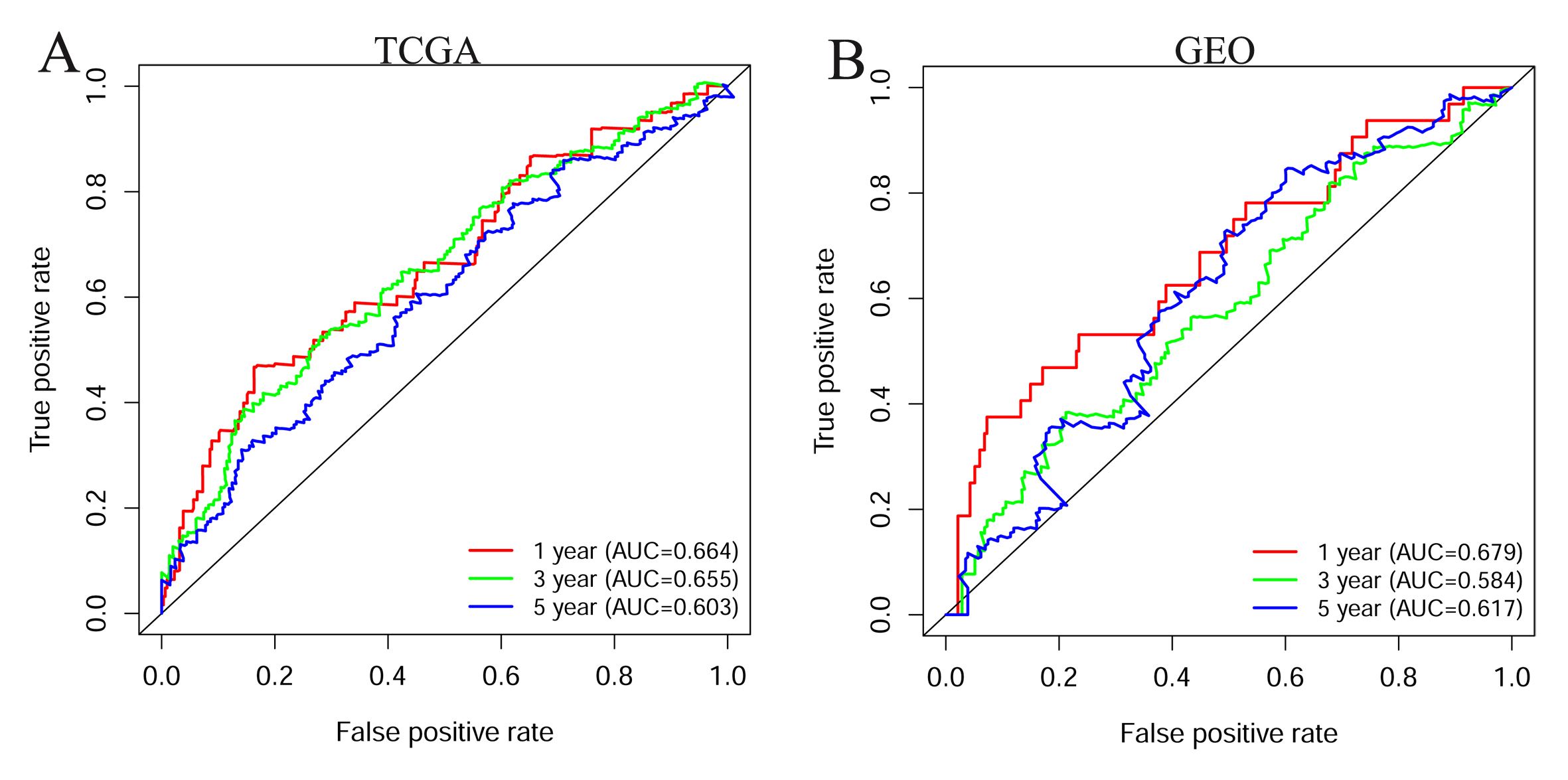

Supplement: Supplementary file 2 [file Image3.JPEG]

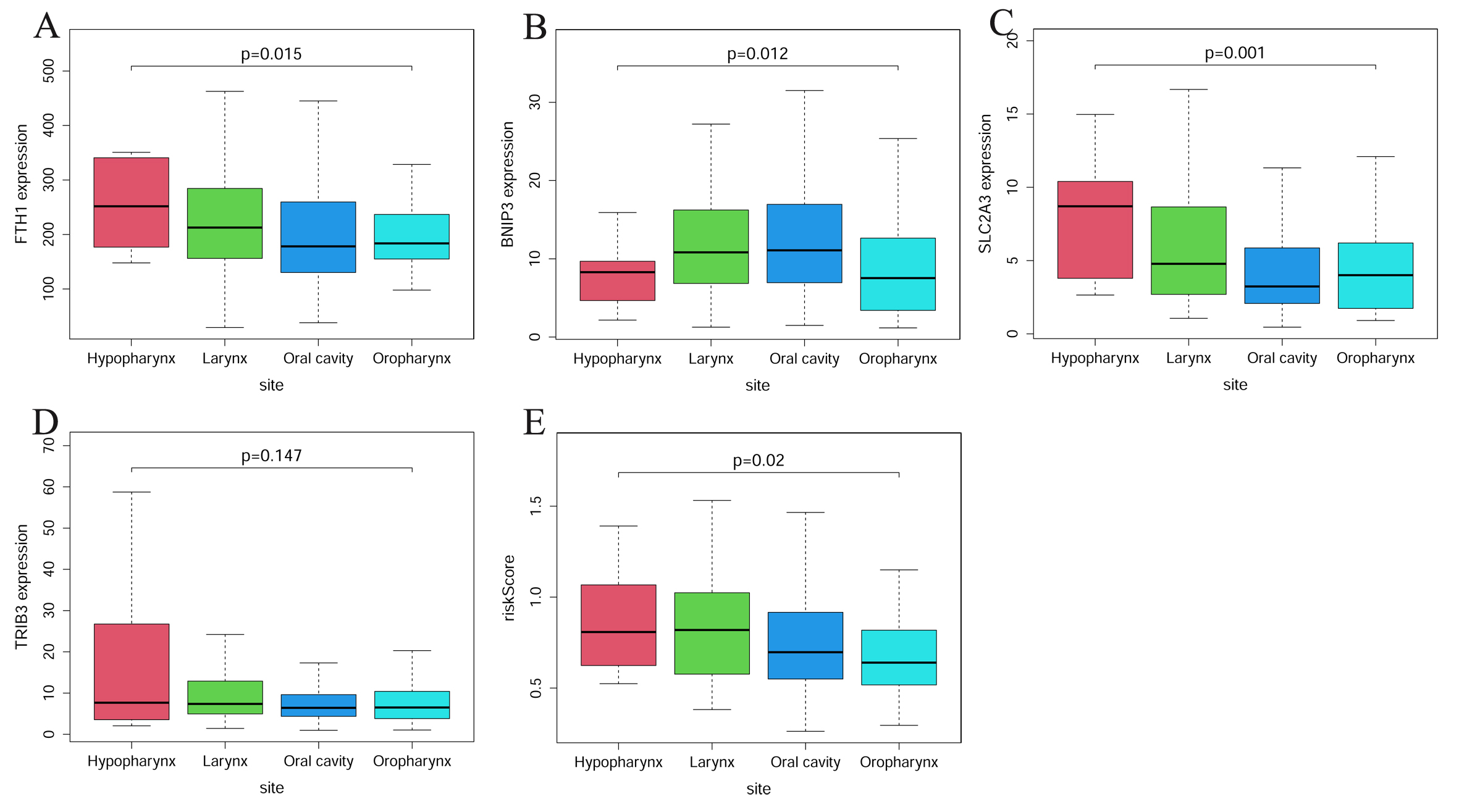

Supplement: Supplementary file 5 [file Image4.JPEG]

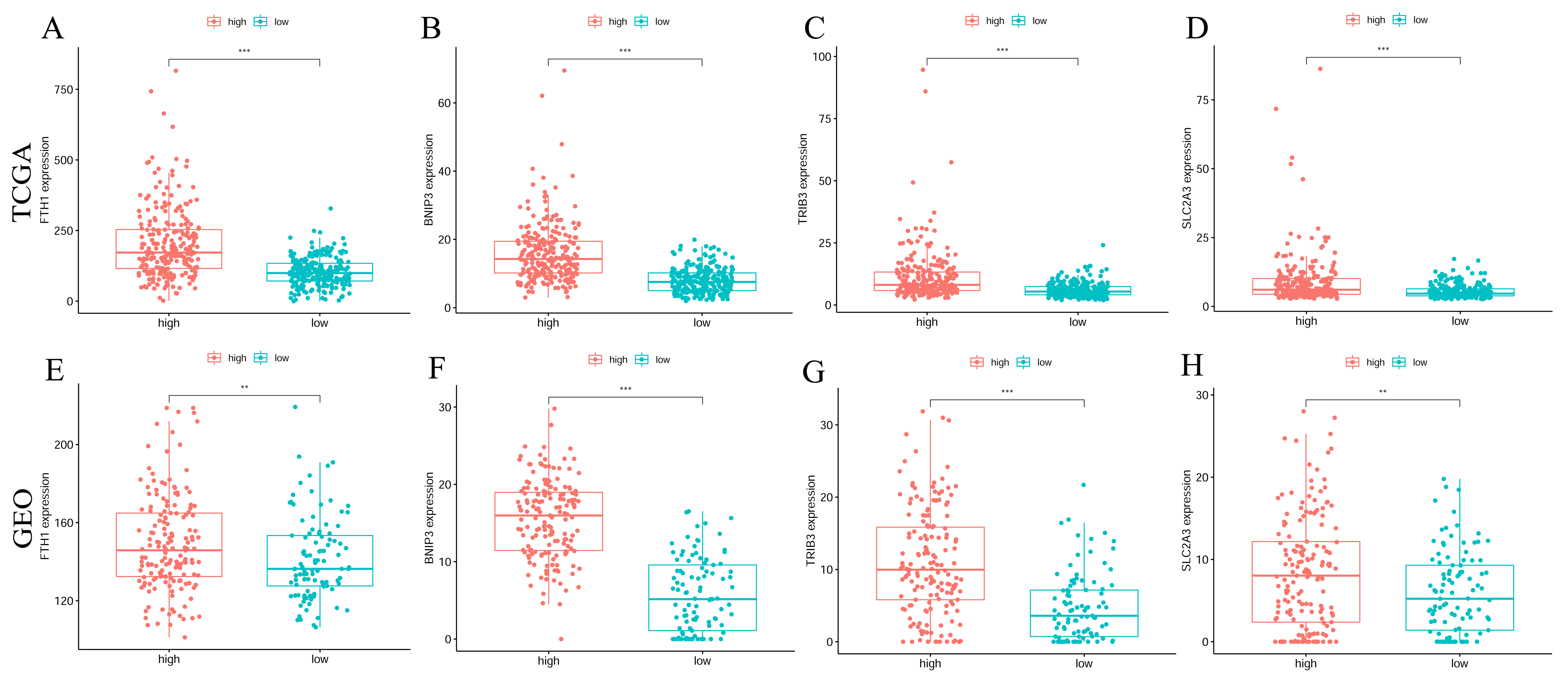

Supplement: Supplementary file 6 [file Image2.JPEG]
