# Supplementary material for: A Ferroptosis-Related Gene Signature for Predicting the Prognosis and Drug Sensitivity of Head and Neck Squamous Cell Carcinoma
Source: Front Genet. 2021 Oct 21;12:755486. doi: 10.3389/fgene.2021.755486 (PMC8566369; doi:10.3389/fgene.2021.755486)
Supplement: Supplementary file 8 [file Table6.DOCX]

<https://www.jianguoyun.com/p/DWOOrQcQyZTeCRjslYcE>

<https://www.jianguoyun.com/p/DcS3PzgQyZTeCRjvlYcE>
