# Supplementary material for: A Ferroptosis-Related Gene Signature for Predicting the Prognosis and Drug Sensitivity of Head and Neck Squamous Cell Carcinoma
Source: Front Genet. 2021 Oct 21;12:755486. doi: 10.3389/fgene.2021.755486 (PMC8566369; doi:10.3389/fgene.2021.755486)
Supplement: Supplementary file 11 [file DataSheet1.docx]

|  | | |  |  |  |
| --- | --- | --- | --- | --- | --- |
| **Supplementary Table 1**. Functions of the prognostic model genes. | | |  | |  |
| **Gene symbol** | **Full name** | **Function** | **Risk coefficient** | | |
| FTH1 | Ferritin Heavy Chain 1 | A vital iron regulatory factor | 0.00141327392932559 | | |
| BNIP3 | BCL2 Interacting Protein 3 | A pro-apoptotic factor | 0.0250779073973851 | | |
| TRIB3 | Tribbles pseudokinase 3 | A fundamental regulator of cell development | 0.01836895046718 | | |
| SLC2A3 | Solute carrier family 2 member 3 | A facilitative glucose transporter | 0.016461171208819 | | |

**Supplementary Table 2.** Relationships between the expression of prognostic model genes and drug sensitivity.

| Gene | Drug | cor | *p* value |
| --- | --- | --- | --- |
| FTH1 | Arsenic trioxide | -0.35598 | 0.005247 |
|  | Carmustine | -0.32514 | 0.01125 |
|  | 6-THIOGUANINE | -0.32036 | 0.012579 |
|  | 6-MERCAPTOPURINE | -0.28628 | 0.026589 |
|  | Tamoxifen | -0.28368 | 0.028057 |
|  | Raltitrexed | -0.28014 | 0.030168 |
|  | Denileukin Diftitox Ontak | -0.2698 | 0.037095 |
| BNIP3 | LDK-378 | -0.40184 | 0.00146 |
|  | Crizotinib | -0.36548 | 0.004085 |
|  | Selumetinib | -0.3597 | 0.004761 |
|  | LEE-011 | -0.35594 | 0.005252 |
|  | PF-06463922 | -0.34107 | 0.007657 |
|  | NMS-E628 | -0.31988 | 0.012721 |
|  | Palbociclib | -0.31237 | 0.015104 |
|  | ARRY-162 | -0.30856 | 0.016456 |
|  | LOXO-101 | -0.29995 | 0.019892 |
|  | Cobimetinib (isomer 1) | -0.29186 | 0.023653 |
|  | Trametinib | -0.27436 | 0.033893 |
|  | 6-THIOGUANINE | -0.27096 | 0.036257 |
|  | gilteritinib | -0.26833 | 0.038176 |
|  | Sunitinib | -0.26641 | 0.039635 |
|  | DECITABINE | -0.26522 | 0.040558 |
|  | Gemcitabine | 0.263536 | 0.041899 |
|  | Carboplatin | 0.264789 | 0.040899 |
|  | Cisplatin | 0.268542 | 0.03802 |
|  | MITOXANTRONE | 0.272154 | 0.03541 |
|  | Sonidegib | 0.27676 | 0.0323 |
|  | Idelalisib | 0.291634 | 0.023768 |
|  | Bleomycin | 0.325752 | 0.011089 |
|  | Zoledronate | 0.365363 | 0.004098 |
|  | Simvastatin | 0.372152 | 0.003411 |
| TRIB3 | Acalabrutinib | -0.33103 | 0.009781 |
|  | Bendamustine | -0.27272 | 0.035013 |
|  | Vismodegib | 0.275159 | 0.033354 |
|  | umbralisib | 0.27889 | 0.03094 |
|  | LOXO-101 | 0.297105 | 0.021151 |
|  | Imiquimod | 0.315641 | 0.014023 |
| SLC2A3 | Carfilzomib | -0.30301 | 0.018604 |
|  | Acetalax | -0.27532 | 0.033248 |
|  | Palbociclib | -0.25436 | 0.049857 |
|  | Trametinib | 0.267128 | 0.039084 |
